# Synthetic CRISPR/dCas9-KRAB system driven by specific *PSA* promoter suppresses malignant biological behavior of prostate cancer cells through negative feedback inhibition of *PSA* expression

**DOI:** 10.1186/s11658-023-00508-y

**Published:** 2023-11-28

**Authors:** Yi Yang, Hongbing Mei, Xiaohong Han, Xintao Zhang, Jianli Cheng, Zhongfu Zhang, Han Wang, Haixia Xu

**Affiliations:** 1 Department of Urology, The First Affiliated Hospital of Shenzhen University, Shenzhen Second People’s Hospital, Shenzhen, China; 2 Department of Medical Oncology, The First Affiliated Hospital of Shenzhen University, Shenzhen Second People’s Hospital, Shenzhen, China

**Keywords:** CRISPR-dCas9-KRAB, Prostate cancer, PSA, Gene therapy

## Abstract

*PSA* is a type of proto-oncogene that is specifically and highly expressed in embryonic and prostate cancer cells, but not expressed in normal prostate tissue cells. The specific expression of prostate-specific antigen (PSA) is found to be related with the conditional transcriptional regulation of its promoter. Clustered regularly interspaced short palindromic repeats (CRISPR)-dCas9-KRAB is a newly developed transcriptional regulatory system that inhibits gene expression by interupting the DNA transcription process. Induction of CRISPR-dCas9-KRAB expression through the *PSA* promoter may help feedback inhibition of cellular *PSA* gene expression via single guide RNA (sgRNA), thereby monitoring and suppressing the malignant state of tumor cells. In this study, we examined the transcriptional activity of the *PSA* promoter in different prostate cancer cells and normal prostate epithelial cells and determined that it is indeed a prostate cancer cell-specific promoter.Then we constructed the CRISPR-dCas9-KRAB system driven by the *PSA* promoter, which can inhibit *PSA* gene expression in the prostate cancer cells at the transcriptional level, and therefore supress the malignant growth and migration of prostate cancer cells and promote their apoptosis in vitro. This study provides a potentially effective anti-cancer strategy for gene therapy of prostate cancer.

## Introduction

The etiology and exact molecular mechanism of primary prostate cancer are not yet fully understood. At present, it is believed that its pathogenesis is a complex process with multiple factors and multiple steps. With the early diagnosis and early treatment of primary prostate cancer, the overall curative effect has been significantly improved [1]. However, even if prostate cancer is radically removed, 60–70% of patients still have metastasis and recurrence within 5 years [2]. Postoperative tumor marker detection and ultrasound examination are used for regular observation to detect the recurrence and metastasis of prostate cancer as soon as possible. Prostate-specific antigen (PSA) is an antigen associated with the prostate [3]. PSA is a single-chain glycoprotein with a molecular weight of 32 kD, secreted by prostate epithelial cells, and it is also a serine protease. Normally, only very low levels of PSA exist in the blood, and an increase in serum PSA concentration indicates pathological changes or trauma to the prostate [4]. A large number of preliminary studies have found that PSA is abundantly expressed in prostate cancer cells and is the most specific marker of prostate cancer [5]. PSA is widely used to diagnose prostate cancer and is a good tumor marker. The US Food and Drug Administration has approved the PSA test as an indicator for the census of men over the age of 50. The specific high expression of PSA in prostate cancer depends on the specific transcriptional activity of its gene promoter. Therefore, the *PSA* gene promoter is often used as a gene therapy for prostate cancer and other tumors. Using the *PSA* promoter to drive suicide gene expression is a commonly used anticancer strategy.

Clustered regularly interspaced short palindromic repeats (CRISPR) is a cluster of short palindromic repeats with regular intervals in bacteria. It is an effective means of finely acquired immunity to resist virus invasion [6]. The general CRISPR-Cas9 system includes a synthetic guide RNA (sgRNA) and Cas9 enzyme. The artificially designed guide RNA replaces the CRISPR RNA (crRNA) in bacteria. The guide RNA in CRISPR-Cas9 consists of two parts: a scaffold used to bind to Cas9 to form a gRNA-Cas9 complex; and a spacer contains an RNA sequence complementary to the target sequence. Since the launch of CRISPR-Cas9, it has completely changed the threshold required for gene editing [7]. A large number of experimental methods and treatment methods based on the CRISPR-Cas9 system have been developed, which has strongly promoted the development of modern medical and biological research [8]. The dead Cas9 (dCas9) is a point mutant of Cas9 nuclease, which loses the ability to cut DNA, but can still bind to it. The fusion of dCas9 and Kox1’s transcriptional repressor domain KRAB (Krüppel-associated box) can interfere with the binding of transcriptase and target sites, and play a role in inhibiting the transcription of targeted genes. This process is called CRISPR interference (CRISPRi) and may have wide applications in gene regulation and disease treatment [9].

The CRISPR-dCas9 system has been widely used in cancer research in recent years. With the gradual clinical application of CRISPR, gene therapy for cancer is likely to be realized in the future [10, 11]. Repairing the genome of cancer cells, such as mutation, chromosomal variation, copy number variation, regulation of tumor cell gene expression, etc. is conducive to the ultimate realization of cancer gene therapy. More importantly, regulating the expression of the CRISPR-dCas9 system using cell transcription-specific promoters enables the recording of transcriptional events that occur within cells over time, ultimately elucidating how molecular events lead to complex cellular behaviors and states [12, 13].

In this study, we have identified the activity pattern of *PSA* promoter in different prostate cancer cells. Then we constructed the CRISPR-dCas9-KRAB system driven by the *PSA* promoter, which can inhibit *PSA* gene expression in cells at the transcriptional level, and therefore suppress the malignant growth and migration of prostate cancer cells and promote their apoptosis. This work provides an effective anticancer strategy for gene therapy of prostate cancer. In the future, the system will be further validated and optimized in experimental models for prostate cancer gene therapy.

## Materials and methods

### Construction of plasmids expressing PSA-dCas9-KRAB and sgRNA-PSA

The promoter region (from −632 to +12 nucleotides) of the *PSA* gene was amplified from the genome of 293 cells by PCR and inserted into the dCas9-KRAB plasmid, which was a gift from Dr. George Church’s lab. The sgRNA targeting and inhibiting the open reading frame (ORF) region of the human *PSA* gene was designed by CRISPR-ERA online software, and its sequence is: 5′-GCTCCCAGCTGCTTTACTAA-3′.

### Cell culture 

Prostate cancer cell lines (LNCap, PC3, AT3B-1, and DU145) and the normal prostate tissue cell line (RWPE1) were purchased from the cell bank of Chinese academy of science, Shanghai. Cells were all cultured in medium RPMI-1640 supplemented with 20% fetal bovine serum (FBS).

### Luciferase reporter assay

The synthesized luciferase reporter plamid was transiently transfected into cells (LNCap, PC3, AT3B-1, DU145, and RWPE1). The whole cell lysate was collected 24 h after transfection and the luciferase activities were measured by the luciferase reporter assay kit (Invitrogen). For data normalization, one reporter generally functions as an experimental reporter and the second as an internal control to account for non-specific experimental variations.

### qRT–PCR

Total RNA was isolated from cells using Trizol agent (Invitrogen) and then reversely transcribed to synthesize cDNA template. Next, quantitative reverse transcription PCR (qRT–PCR) was performed by using a cDNA template and specific PCR primers. The change of the fluorescent signal was used to detect the change in the amount of the amplification product in each cycle of the PCR amplification reaction in real time, and finally the starting template was accurately quantitatively analyzed.

### Cell growth assay

The MTT assay was used for determining cell proliferation 48 h after transfection according to the supplier’s instructions. Prostate cancer cells in the logarithmic growth phase were cultured in 96-well plates, and 5000 cells were seeded per well. The light absorption value was measured with an enzyme-linked immunosorbent assay (ELISA) at 570 nm wavelength, which indirectly reflects the number of living cells.

For colony formation assay, cells were seeded into each well of a six-well plate and incubated for 2 weeks. Cell colonies were fixed with 4% PFA and then stained with 1% crystal violet for 15 min. Finally, the plate was rinsed gently with distilled water. A BX51 microscope (Olympus, Tokyo, Japan) was used to count stained colonies.

### Cell migration assay

Migration of human prostate cancer cell lines was assessed using a cell scratch assay. A marker was used to draw two parallel lines on the back of the six-well plate before cell seeding, and cells are seeded into the six-well plate after digestion. When the cells cover the bottom of the plate, a 10 μl pipette tip was used to gently draw lines on the plate, and the width of each scratch should be as close to the same as possible. After rinsing the plate three times with phosphate-buffered saline (PBS) buffer to remove cell debris from scratches, the cells were photographed. Finally, pictures were collected for analysis, and migration rate was quantified by dividing the change in wound width by the time spent in migration.

Transwell migration assay was performed by using uncoated membranes of Transwell chambers. Briefly, cells were suspended in serum-free medium. A total of 200 µl of cell suspension was added to the upper chamber; 700 µl of culture medium (10% FBS) was added to the lower chamber. Next, cells were grown in a 37 °C incubator. After 24 h, cells that passed through the membrane were fixed with 4% PFA and then stained with 1% crystal violet to remove cells on the other side of the membrane. Stained cells were counted in five random fields under a BX51 microscope.

### Cell apoptosis assay

Cell apoptosis was determined by ELISA, which is based on a fluorescent immunosorbent enzyme reaction, using the Caspase-3 Activity Assay kit (Beyotime Shanghai, China). Briefly, whole cell lysates were added to 96-well plates and incubated with 2 mM caspase-3 substrate for 4 h at 37 °C. Then, the absorbance was determined at 405 nm using a microplate reader. Apoptosis was also detected using Annexin V-PI Apoptosis Detection Kit (A211, Vazyme, Nanjing, China). Transfected prostate cancer cells were harvested and washed with PBS, then cells were resuspended in 100 µl of Annevix Binding Buffer and incubated with 5 µl of Annevix FITC and 5 µl of propidium iodide (PI) for 15 min. Finally, apoptosis was detected after adding 150 µl of Annevix Binding Buffer.

### Statistical analysis

Data are shown as the mean ± one standard deviation (SD) and analyzed by a *t*-test. A *P* < 0.05 was considered statistically significant. All the processes of statistical analysis were performed using IBM SPSS Statistics Version 20 software.

## Results

### *PSA* promoter specifically induces reporter gene expression in prostate cancer cell lines

**Fig. 1:**
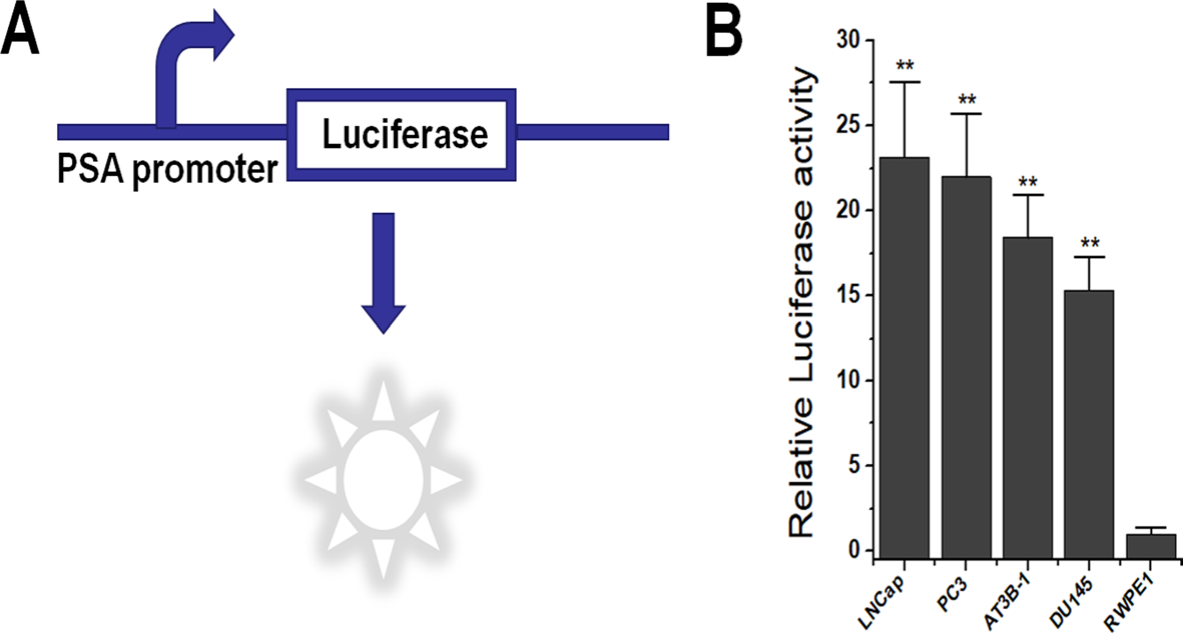
The reporter gene expression driven by *PSA* promoter in prostate cancer lines. **A** Construction of a luciferase reporter gene expression plasmid driven by the *PSA* promoter, with an additional constitutive promoter driving luciferase as an internal reference, which is not depicted in the figure. **B** Luciferase activity detected in prostate cancer cell lines and the normal prostate cell line. **P < 0.01 (Student’s *t*-test, *n* = 3 replicates)

To confirm the specific transcriptional activity of the *PSA* gene promoter in prostate cancer, we constructed a dual-luciferase reporter gene expression plasmid, in which one luciferase gene was driven by the *PSA* promoter (Fig. 1A). By transiently transfecting constructed plasmids into prostate cancer cell lines (LNCap, PC3, AT3B-1, and DU145) and normal prostate tissue cell lines (RWPE1), we found that luciferase only has a relatively high expression activity in prostate cancer cells at 48 h after transfection (Fig. 1B). This confirmed that the *PSA* promoter is a prostate cancer-specific promoter, which was consistent with the results of previous studies [3–5].

### *PSA* promoter specifically drives the expression of dCas9-KRAB gene in prostate cancer cells

**Fig. 2:**
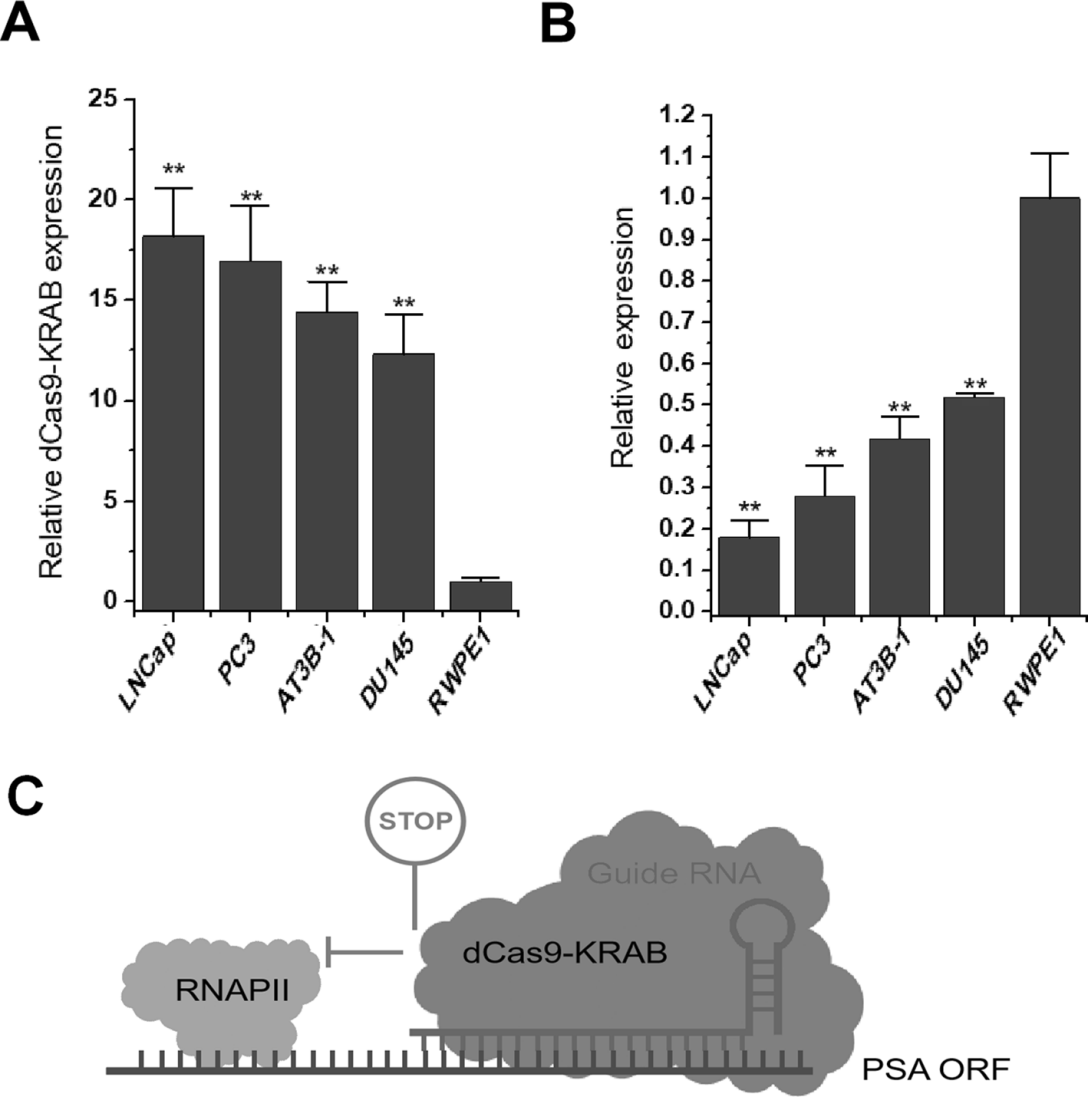
dCas9-KRAB gene expression in prostate cancer cells. **A** RNA expression levels of dCas9-KRAB in four prostate cancer cell lines and one prostate cell line. **B** RNA expression levels of PSA mRNA in four prostate cancer cell lines and one prostate cell line. **C** dCas9-KRAB/sgRNA targeting the PSA ORF region locally forms heterochromatin and blocks the sliding of RNA polymerase on the DNA strand. **P < 0.01 (Student’s *t*-test, *n* = 3 replicates)

After confirming the specificity of the *PSA* promoter in prostate cancer, we further constructed a dCas9-KRAB expression plasmid driven by the *PSA* promoter. After treating prostate cancer cell line and normal prostate cell line respectively, we detected the expression level of dCas9-KRAB mRNA at 48 h after transfection. The results showed that only significant dCas9-KRAB expression was detected in prostate cancer cells (Fig. 2A). At the same time, we designed gRNAs that target the *PSA* gene. After 48 h of transfection of different cells, it was found that it can specifically inhibit the expression level of PSA mRNA in prostate cancer cells (Fig. 2B). The mechanism of dcas9-KRAB system-mediated *PSA* gene expression using sgRNA against ORF of *PSA* gene was shown in Fig. 2C.

These results suggest that the *PSA* promoter can indeed overexpress dCas9-KRAB specifically in prostate cancer cells.

### CRISPR-dCas9-KRAB driven by *PSA* promoter efficiently and specifically inhibits cell proliferation and migration in prostate cancer

**Fig. 3:**
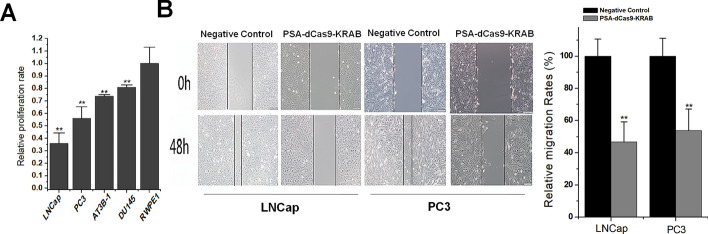
*PSA* promoter-driven CRISPR-dCas9-KRAB inhibits the proliferation and migration of prostate cancer cells. **A** MTT experiment results found that PSA-dCas9-KRAB significantly inhibited the viability of prostate cancer cells. **B** Cell scratch experiment results found that PSA-dCas9-KRAB significantly inhibited the migration rate of prostate cancer cells. **P < 0.01 (Student’s *t*-test, *n* = 3 replicates)

After successfully constructing the dCas9-KRAB expression plasmid by the *PSA* promoter and verifying its expression effect, we tested the level of cell malignancy affected by *PSA* feedback inhibition. After treating prostate cancer cell line and normal prostate cell line respectively, we detected the cell proliferation level at 48 h after transfection. MTT experiment results found that PSA-dCas9-KRAB significantly inhibited the viability of prostate cancer cells, and did not affect the proliferation of normal cells (Fig. 3A). Images were acquired at 0 and 48 h in cell scratch assay, and the results showed that PSA-dCas9-KRAB also reduced the relative migration rates of several transfected prostate cancer cell lines compared with that transfected with the the non-target gRNA and dCas9-KRAB expression control (Fig. 3B). This result suggests that PSA-dCas9-KRAB specifically and efficiently inhibits the proliferation and migration of prostate cancer cells and has a potential anticancer activity.

**Fig. 4:**
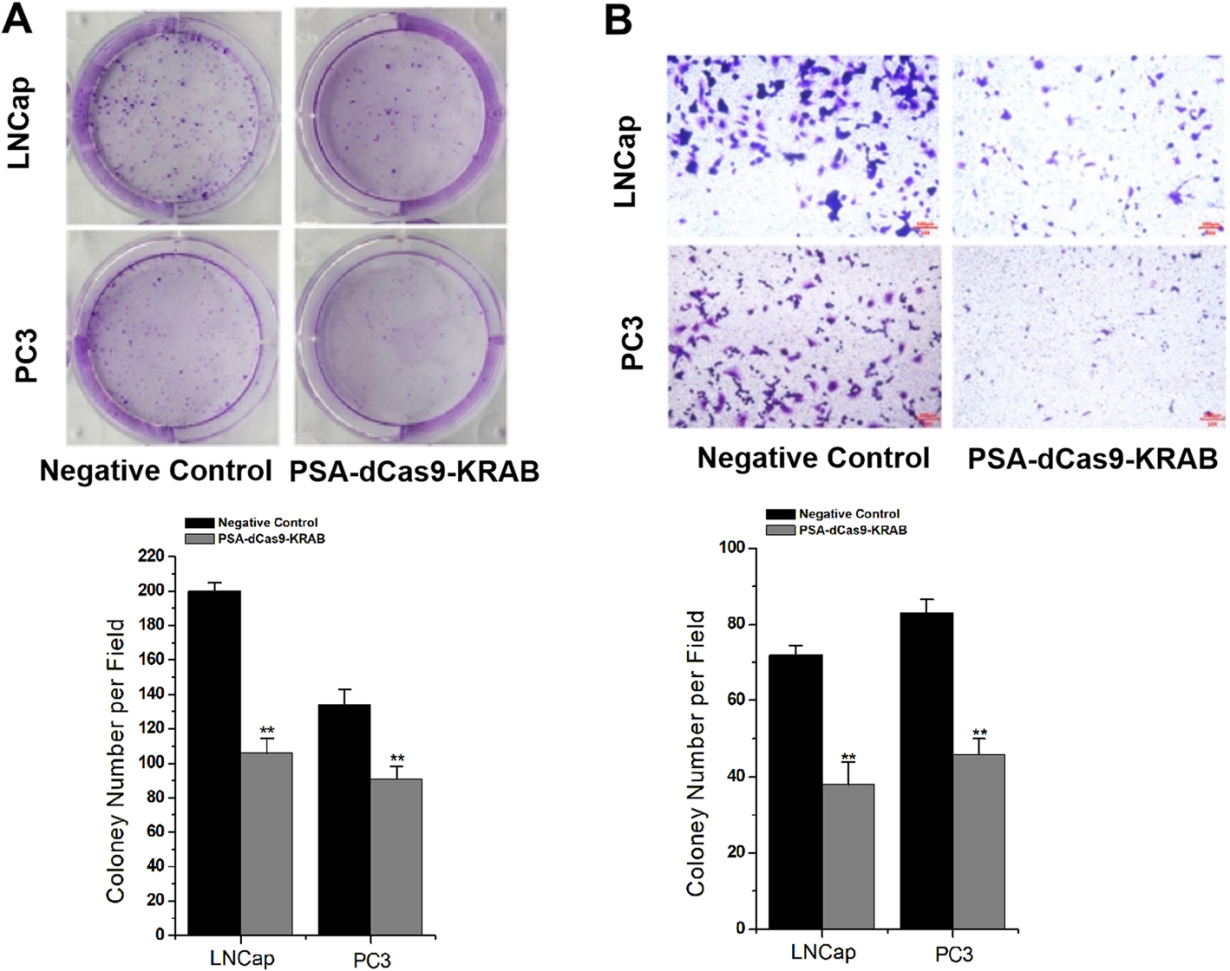
*PSA* promoter-driven CRISPR-dCas9-KRAB suppresses the colony-forming ability and transwell transmembrane migration ability of prostate cancer cells. **A** The colony formation assay revealed that PSA-dCas9-KRAB significantly inhibited the colony-forming ability of prostate cancer cells. **B** Transwell assay revealed that PSA-dCas9-KRAB significantly inhibited the migration number of prostate cancer cells. **P < 0.01 (Student’s *t*-test, *n* = 3 replicates)

To further confirm the reproducibility of the above results, we used colony formation assay and transwell assay to detect the level of cell proliferation and migration 48 h after plasmid transfection of prostate cancer cell lines LNCap and PC3. The results of the colony formation assay found that PSA-dCas9-KRAB significantly inhibited the clonogenic ability of prostate cancer cells (Fig. 4A). Images were acquired at 48 h in a transwell assay and showed that PSA-dCas9-KRAB also reduced the number of migrations in both transfected prostate cancer cell lines compared with cell lines transfected with the non-target gRNA and dCas9-KRAB expression controls (Fig. 4B).

### CRISPR-dCas9-KRAB driven by *PSA* promoter efficiently and specifically promotes the apoptosis of prostate cancer cells

**Fig. 5:**
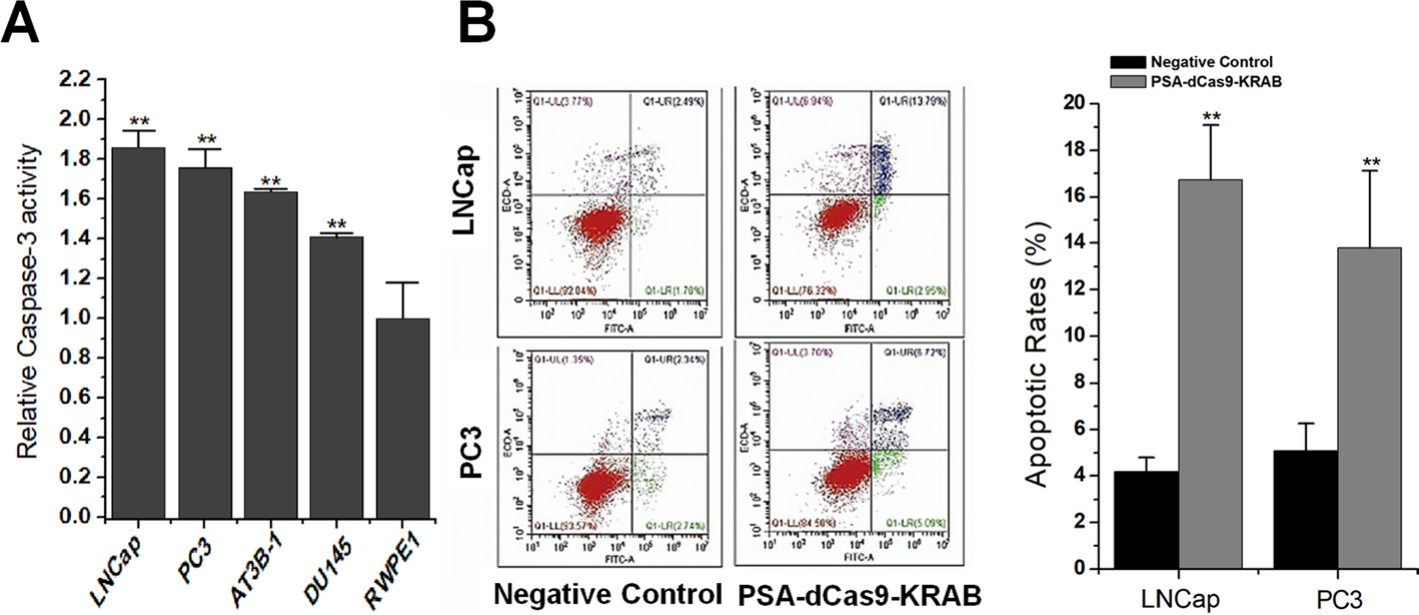
*PSA* promoter-driven CRISPR-dCas9-KRAB promotes the apoptosis of prostate cancer cells. **A** ELISA experiment results found that PSA-dCas9-KRAB significantly promoted the apoptosis of prostate cancer cells. **B** Flow cytometry analysis found that PSA-dCas9-KRAB significantly promoted the apoptosis of prostate cancer cells. **P < 0.01 (Student’s *t*-test, *n* = 3 replicates)

After verifying the potential anticancer activity of PSA-dCas9-KRAB, we further tested the level of apoptosis. After treating prostate cancer cell line and normal prostate cell line, respectively, we detected the apoptosis level of the cells 48 h after transfection. ELISA results showed that PSA-dCas9-KRAB significantly promoted the apoptosis of prostate cancer cells by increasing the caspase-3 activity, and did not affect normal cells (Fig. 5A). To further confirm this result, we also performed flow cytometry analysis. The data showed that ratios of apoptotic cells were increased in cells transfected with PSA-dCas9-KRAB (Fig. 5B). This result suggests that PSA-dCas9-KRAB specifically and efficiently promotes the apoptosis of prostate cancer cells and has significant anti-cancer activity.

## Discussion

Cancer is a major killer that threatens human health and life. Traditional treatments, such as radiation and chemotherapy, destroy cancer cells by creating DNA double-strand breaks in their DNA [14]. But because the two therapies cannot distinguish cancer cells from normal cells, when they are administered, they indiscriminately kill healthy cells with inevitable side effects. Many studies have long searched for an ideal tumor therapy that selectively targets only cancer cells, leaving normal cells unaffected [15]. Targeted therapy, which has emerged in recent years, is a gene therapy method based on cellular molecular water, which can identify gene expression changes specific to tumor cells. By targeting the already defined carcinogenic sites, different targeted drugs are used to block the signal transduction pathways of cancer cell growth and reproduction, thereby killing cancer cells [16].

There are many studies on cancer gene therapy that used tumor-specific promoters such as promoters of *TERT* and *PSA* genes [17]. Most of them used tumor-specific promoters to drive the expression of a suicide gene to fight against cancer. However, suicide genes do not always work effectively in cells to drive cancer cell death. Cancer cells can become resistant, not only to a variety of chemotherapeutic drugs and molecular targeted drugs, but also to the expression and effect of suicide genes [18]. Achieving maximum inhibition of cancer cells requires targeting key molecules that are actively expressed in tumors. The biggest advantage of CRISPR-dCas9-KRAB is that it can flexibly target the gene of interest [19]. Taking into account that *PSA* has a high level of expression in prostate cancer cells, an anticancer system that feedback-inhibits *PSA* could be constructed if gRNA is used to target the expression of *PSA*. In this way, the highly expressed PSA signal of prostate cancer cells can be used to inhibit their own expression levels in turn through a built-in negative feedback system, thereby inhibiting tumors specifically and efficiently.

Under the guidance of the above ideas, in this work, we used the *PSA* promoter to specifically drive the expression of dCas9-KRAB in prostate cancer cells, and then designed gRNA to inhibit the expression of *PSA*. To prevent gRNA from inhibiting the transcription of the *PSA* promoter on the plasmid, we specially designed the target of gRNA to the ORF region of the *PSA* gene. dCas9-KRAB generates truncated PSA mRNA by inhibiting the slippage of RNA polymerase on the DNA strand, which eventually leads to the degradation of PSA mRNA and the inactivation of cellular PSA. The system works only in prostate cancer cells and it not only inhibits the proliferation and migration of prostate cancer cells, but also promotes their apoptosis. The possible molecular mechanism may be related to the regulation of the expression of antiangiogenic genes downstream of PSA. So it has a safe and controllable anticancer effect.

This work presents a very specific and highly effective anticancer system that hardly affects normal cells, and has a very good clinical application prospect. It provides a safe and controllable method for specifically targeting cancer cells, offering a potential solution to the long-standing challenge of distinguishing between cancerous and healthy cells in treatment. This technology holds great promise for improving the clinical management of cancer and underscores the potential of CRISPR-based approaches in the field of tumor gene therapy.

Of course, this study also has certain limitations. The overall transcriptional activity of the *PSA* promoter used is relatively weak. This situation is common with tumor-specific promoters, which are often epigenetically silenced in normal cells [20]. In the future, how to further improve the transcriptional activity of the *PSA* promoter in prostate cancer, and then apply the device to in vivo experiments, is a matter worth considering. Tumor-specific enhancer elements can be used in combination with the *PSA* promoter, which may ensure its targeting specificity while enhancing its transcriptional activity [21]. Another possible approach is to analyze whether the *PSA* promoter is bound by transcriptional repressors [22]. The transcriptional activity can also be enhanced by inhibiting the expression of transcriptional repressors. But it must be ensured that this does not affect its transcriptional specificity. The effectiveness of different methods remains to be further tested and validated. Overall, like other recent similar works [23–25], this study once again demonstrates the potential application of the CRISPR system for tumor gene therapy.

## Conclusion

Our study provides a potentially effective anticancer strategy based on CRISPR-dCas9-KRAB system for gene therapy of prostate cancer.

## Data Availability

Further inquiries can be directed to the corresponding authors.
